# The cellular choreography of brain aging: a neuroimmune network perspective

**DOI:** 10.3389/fnagi.2026.1848642

**Published:** 2026-05-22

**Authors:** Ludmila Müller, Svetlana Di Benedetto, Viktor Müller

**Affiliations:** Max Planck Institute for Human Development, Center for Lifespan Psychology, Berlin, Germany

**Keywords:** brain aging, cellular aging trajectories, epigenetic regulation, metabolic reprogramming, neuroimmune interactions, neuroimmune networks, neuroinflammation, systems neuroscience

## Abstract

Brain aging is increasingly recognized as a heterogeneous and systems-level process involving dynamic interactions among neuronal, glial, vascular, and immune-associated cell populations. Recent advances in single-cell and spatial omics technologies have revealed diverse cellular aging trajectories, region-specific vulnerabilities, and extensive remodeling of intercellular communication networks across the aging brain. These findings challenge reductionist views of aging and emphasize the importance of understanding how cellular interactions collectively shape neural function and resilience. In this mini-review, we examine brain aging from a neuroimmune and network-based perspective, focusing on how coordinated interactions among neurons, glial cells, and vascular elements regulate tissue homeostasis during aging. We propose the concept of a cellular choreography as a system-level framework for understanding how dynamic interactions among neurons, glial cells, vascular elements, and neuroimmune signaling pathways shape brain aging. Rather than acting independently, these cellular systems continuously coordinate metabolic support, inflammatory responses, synaptic activity, and vascular regulation across the neurovascular unit. Aging progressively alters these interactions, contributing to synaptic dysfunction, glial reactivity, blood–brain barrier impairment, and chronic low-grade inflammation. We further discuss shared molecular programs—including mitochondrial dysfunction, impaired proteostasis, cellular senescence, DNA damage responses, and epigenetic remodeling—that influence multiple cell populations and propagate dysfunction across interconnected cellular networks. Finally, we highlight emerging longitudinal, multiomic, and stem cell–based approaches that are beginning to map cellular aging trajectories across multiple biological scales. Integrating these approaches within systems-level frameworks may improve the identification of early biomarkers and therapeutic targets aimed at promoting resilient and healthy brain aging.

## 1 Introduction: brain aging as a multi-cellular network process

Brain aging is increasingly recognized as a dynamic and multifactorial process that emerges from the interactions of diverse cellular populations within the central nervous system (CNS) (De Domenico, 2017). Neurons, glial cells—including astrocytes, microglia, and oligodendrocytes—and vascular components together form highly interconnected cellular systems that maintain neural homeostasis and support cognitive function throughout life (Müller et al., 2025a). With advancing age, these interactions undergo gradual yet profound alterations, affecting synaptic communication, metabolic support, immune surveillance, and neurovascular coupling. Rather than reflecting the isolated decline of individual cell types, brain aging arises from coordinated changes across multiple cellular compartments that collectively reshape the functional architecture of the brain (Müller et al., 2025b).

A central feature of this process is the pronounced heterogeneity that characterizes aging across cell types, brain regions, and even individual cells within the same population. Neuronal subtypes exhibit differential vulnerability to metabolic stress and synaptic dysfunction, while glial cells display diverse reactive states that can shift between protective and pro-inflammatory phenotypes, contributing to neuroinflammaging (Table 1). Similarly, vascular elements of the neurovascular unit (NVU) show region-specific alterations that influence blood–brain barrier (BBB) integrity and cerebral perfusion (Tsintzou et al., 2025; Morrison and Baxter, 2012; Pacca-Corrêa et al., 2026; Sun et al., 2025; Song and Dityatev, 2018; Hu and Tao, 2024; Müller and Di Benedetto, 2024). This cellular diversity suggests that brain aging unfolds along distinct trajectories that vary across neural circuits and anatomical regions.

**Table 1:** Selected concepts related to brain aging and neuroimmune interactions.

Term	Definition
Neuroinflammaging	Chronic low-grade neuroinflammatory state associated with aging, characterized by persistent activation of immune and glial signaling pathways in the brain.
Microglial priming	Age-related state in which microglia exhibit heightened sensitivity and exaggerated inflammatory responses to subsequent stimuli.
Reactive astrocytes	Astrocytes undergoing molecular and functional changes in response to aging, injury, or inflammation, often involving altered inflammatory and metabolic signaling.
Cellular senescence	Cellular state characterized by stable cell-cycle arrest and secretion of pro-inflammatory and stress-associated factors.
Neurovascular unit (NVU)	Functional multicellular complex composed of endothelial cells, pericytes, astrocytes, neurons, microglia, and vascular smooth muscle cells regulating cerebral homeostasis.
Blood–brain barrier (BBB)	Specialized vascular interface formed primarily by endothelial cells and supporting perivascular structures that regulates molecular exchange between blood and the CNS.
Cellular choreography	Conceptual framework describing the dynamic coordination and interaction of neuronal, glial, vascular, and immune-associated cellular networks during brain aging.

In this context, brain aging can be viewed as a form of cellular choreography, in which neuronal, glial, vascular, and immune-associated cells dynamically interact within highly interconnected signaling networks. These coordinated interactions regulate synaptic activity, metabolic exchange, vascular homeostasis, and inflammatory responses that are essential for maintaining neural function. With advancing age, alterations in cellular communication, metabolic balance, and immune regulation progressively reshape these network interactions, contributing to circuit dysfunction and increased vulnerability to neurodegenerative disease. Understanding this coordinated cellular choreography across molecular, cellular, and systems levels is therefore essential for deciphering the mechanisms that drive brain aging and for identifying strategies that support neural resilience across the lifespan.

## 2 Cellular heterogeneity and vulnerability in the aging brain

Aging of the brain does not occur uniformly across cellular populations or anatomical regions. Instead, accumulating evidence indicates that distinct cell types follow divergent aging trajectories, characterized by cell-specific transcriptional, metabolic, and functional changes. These trajectories reflect intrinsic cellular programs as well as the influence of local microenvironments and intercellular signaling networks (Müller et al., 2025a; Seguin et al., 2023; Lovinger, 2008). Consequently, vulnerability to aging-related dysfunction varies substantially across neuronal and non-neuronal populations, shaping the spatial and functional landscape of brain aging (Müller et al., 2025b; Lindenberger, 2014).

Neurons exhibit pronounced heterogeneity in their susceptibility to aging-associated stressors. Certain neuronal subtypes—including long-range projection neurons and metabolically demanding excitatory neurons—appear particularly vulnerable to mitochondrial dysfunction, impaired proteostasis, and synaptic alterations (Burke and Trudeau, 2022; Pass et al., 2021). Transcriptomic analyses have revealed age-associated shifts in gene expression linked to synaptic maintenance, calcium signaling, and energy metabolism. For example, single-nucleus RNA sequencing of the human prefrontal cortex demonstrated subtype-specific transcriptional changes in excitatory neurons, including altered expression of genes involved in synaptic transmission and mitochondrial pathways (Li et al., 2022; Tran et al., 2021). Similarly, studies in the mouse brain have shown that neuronal populations display heterogeneous transcriptional aging signatures, suggesting that neuronal identity strongly influences cellular aging trajectories (Ximerakis et al., 2019).

Glial populations also exhibit marked heterogeneity in aging responses. Microglia undergo transcriptional remodeling characterized by increased expression of inflammatory mediators, immune receptors, and genes associated with phagocytic activity (Badimon et al., 2020; Müller and Di Benedetto, 2025a). Single-cell analyses have identified distinct microglial states emerging with age, including populations associated with inflammatory priming and altered immune surveillance (Ali et al., 2025; Hammond et al., 2019). Astrocytes likewise display age-dependent changes in gene expression related to metabolic support, neurotransmitter regulation, and inflammatory signaling, indicating that glial aging involves shifts along a spectrum of reactive phenotypes rather than a single uniform state (Pacca-Corrêa et al., 2026; Pessoa et al., 2026). Oligodendrocyte lineage cells show altered differentiation dynamics and myelin maintenance with age, suggesting that white matter integrity may also reflect cell-type–specific aging processes (Lopez-Muguruza and Matute, 2023; Marques et al., 2016).

Regional differences further contribute to the heterogeneous landscape of brain aging. Comparative transcriptomic studies have revealed that some brain regions—such as the hippocampus, prefrontal cortex, and substantia nigra—display earlier or more pronounced molecular and cellular alterations than others (Ham and Lee, 2020; Lupo et al., 2019). These region-specific vulnerabilities likely arise from differences in neuronal composition, metabolic demand, connectivity patterns, and exposure to systemic factors. For instance, spatial transcriptomic profiling has shown that aging-associated gene expression changes can vary significantly across cortical layers and hippocampal subfields, highlighting the importance of local microenvironments in shaping cellular responses to aging (Huuki-Myers et al., 2024; Barberis and Xie, 2026). Together, these technologies are transforming our understanding of how aging unfolds across cellular networks, uncovering region-specific gene expression programs and cell–cell communication pathways that contribute to brain aging (Barberis and Xie, 2026; Zhang D. et al., 2025).

Understanding this diversity is essential for identifying the mechanisms that drive vulnerability or resilience in different cell populations. In the following sections, we therefore examine the aging trajectories of major cellular components of the brain—including neurons, glial cells, and vascular elements—highlighting how their interactions collectively shape the network-level processes underlying brain aging.

## 3 Neuronal aging: synaptic and metabolic decline

**Figure 1:**
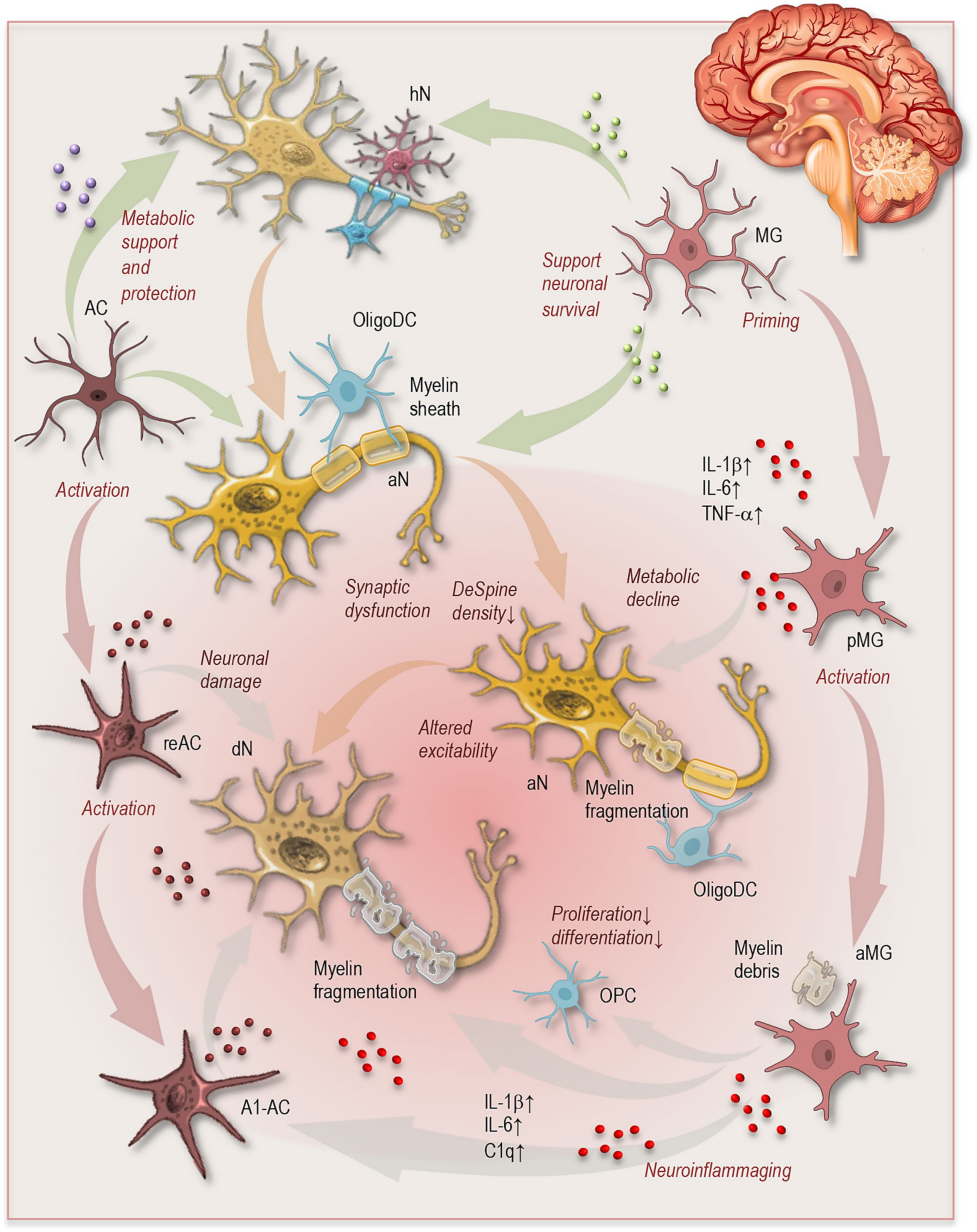
Cellular circuits during neuronal and glial aging. Simplified schematic illustrating dynamic interactions among neurons, astrocytes, microglia, and oligodendrocyte lineage cells during brain aging. In the healthy state (upper panel), astrocytes provide metabolic support and protection to neurons, oligodendrocytes maintain myelin integrity, and microglia contribute to homeostasis and neuronal survival. With aging (lower panel), neuronal metabolic decline, synaptic dysfunction, altered excitability, and dendritic spine loss emerge. Concurrently, astrocytes and microglia adopt reactive states, characterized by increased inflammatory signaling and priming of microglia, contributing to inflammaging. Oligodendrocyte lineage cells exhibit reduced differentiation and impaired myelin maintenance, leading to myelin fragmentation and accumulation of myelin debris. These processes interact through interconnected signaling loops, illustrating how age-related cellular changes propagate across neuroglial networks and contribute to altered circuit function. hN, healthy neuron; aN, aging neuron; dN, degenerating neuron; DeSpine, dendritic spine; MG, microglia; pMG, primed microglia; aMG, activated microglia; AC, astrocyte; reAC, reactive astrocyte; A1-AC, neurotoxic astrocyte; OligoDC, oligodendrocyte; OPC, oligodendrocyte precursor cell; IL, interleukin; TNF, tumor necrosis factor.

Neurons are among the most metabolically demanding and long-lived cells in the brain, making them particularly vulnerable to the cumulative effects of aging (Figure 1). Although widespread neuronal loss is not a defining feature of normal aging, numerous studies indicate that functional and molecular alterations in neuronal physiology emerge progressively over time. These changes include synaptic dysfunction, impaired mitochondrial function, and altered neuronal excitability, which together contribute to age-related changes in neural circuit performance and cognitive function (Lindenberger, 2014; Lee and Kim, 2022; Abrous et al., 2026).

### 3.1 Synaptic dysfunction

One of the most consistent features of neuronal aging is the gradual decline in synaptic integrity and plasticity. Structural and functional changes at synapses include reductions in dendritic spine density, alterations in synaptic protein expression, and impaired neurotransmission (Figure 1). These changes can disrupt synaptic connectivity and reduce the efficiency of information transfer across neural circuits (Abrous et al., 2026; Barrantes, 2024; Di Benedetto et al., 2017).

Experimental studies in rodents and human have demonstrated that aging is associated with reduced long-term potentiation (LTP) and altered synaptic plasticity, particularly in the hippocampus, a region critical for learning and memory (Barnes, 1994; Burke and Barnes, 2006; Villarreal et al., 2002). Age-dependent reductions in dendritic spine density and synaptic contacts have also been reported in cortical and hippocampal neurons, suggesting that synaptic remodeling contributes to functional decline in aging neural circuits (Morrison and Baxter, 2012; Bloss et al., 2011; Radulescu et al., 2025).

Human studies provide converging evidence for synaptic vulnerability during aging. Postmortem and neuroimaging studies further support the presence of age-associated synaptic alterations, including reductions in synaptic density markers, changes in dendritic spine morphology, and altered functional connectivity within hippocampal and cortical networks associated with cognitive decline (Morrison and Baxter, 2012). Postmortem analyses have revealed age-associated decreases in synaptic markers, including synaptophysin and PSD-95, in cortical regions involved in cognition (Henstridge et al., 2015; Head et al., 2009). Moreover, imaging and electrophysiological studies suggest that changes in synaptic connectivity and plasticity may contribute to age-related cognitive decline even in the absence of overt neurodegeneration (Morrison and Baxter, 2012; Radulescu et al., 2025).

### 3.2 Mitochondrial impairment and metabolic stress

Mitochondrial dysfunction represents another key feature of neuronal aging. Neurons rely heavily on oxidative phosphorylation to sustain synaptic transmission and ion homeostasis, making mitochondrial integrity essential for neuronal survival and function. With advancing age, mitochondria exhibit reduced respiratory capacity, increased production of reactive oxygen species (ROS), and impaired mitochondrial dynamics (Pass et al., 2021; Bondy, 2024; Srivastava, 2017).

Experimental evidence indicates that aging neurons accumulate mitochondrial DNA mutations and display deficits in mitochondrial biogenesis and quality control pathways. These alterations can compromise ATP production and increase oxidative stress, thereby affecting synaptic transmission and neuronal resilience. In mouse models, impaired mitochondrial transport along axons has also been shown to disrupt synaptic energy supply and contribute to synaptic dysfunction (Pass et al., 2021; Bondy, 2024; Zong et al., 2024).

Evidence from human studies similarly implicates mitochondrial decline in neuronal aging. Analyses of human cortical tissue have revealed age-related changes in mitochondrial gene expression and reductions in respiratory chain activity (Theurey and Pizzo, 2018; Ojaimi et al., 1999). In addition, neuroimaging studies indicate that metabolic activity declines in specific brain regions with age, including the prefrontal cortex and hippocampus, consistent with impaired neuronal energy metabolism (Zhang X. et al., 2025; Deery et al., 2023).

### 3.3 Altered neuronal excitability

Age-related changes in neuronal physiology also include alterations in intrinsic excitability and calcium homeostasis. Electrophysiological recordings from aged hippocampal neurons have demonstrated increased calcium influx and prolonged afterhyperpolarization currents, which can modify firing patterns and impair synaptic plasticity (Kumar et al., 2009). These changes may arise from altered ion channel expression, disrupted calcium buffering, or mitochondrial dysfunction.

Such shifts in excitability can have important consequences for network dynamics, affecting synaptic integration, signal fidelity, and the balance between compensatory activity and circuit instability during aging. In some circuits, aging is associated with reduced signal-to-noise ratios and impaired synaptic integration, whereas in others, compensatory increases in neuronal activity may occur (Cabeza et al., 2002; Heuninckx et al., 2008; Müller et al., 2019). Functional MRI studies in aging humans have frequently reported increased bilateral recruitment of frontal and parietal cortical regions during memory and executive tasks, a phenomenon interpreted as compensatory network reorganization aimed at preserving cognitive performance despite declining neural efficiency. Mechanistically, such adaptations may involve altered excitatory–inhibitory balance, redistribution of network activity, and increased functional connectivity among higher-order association regions (Cabeza et al., 2002).

### 3.4 Consequences for neural circuits

The combined effects of synaptic dysfunction, metabolic decline, and altered excitability ultimately influence the organization and performance of neural circuits. Aging-related disruptions in neuronal signaling can impair communication across distributed brain networks that support cognitive functions such as memory, attention, and executive control (Navakkode and Kennedy, 2024).

At the systems level, these alterations may contribute to reduced network flexibility and decreased efficiency of information processing. However, the aging brain also exhibits considerable plasticity, and compensatory mechanisms—including synaptic remodeling and recruitment of alternative circuits—may help sustain cognitive function in many individuals (Müller et al., 2025a,c; Abrous et al., 2026; Cabeza et al., 2002; Navakkode and Kennedy, 2024).

Together, these findings highlight that neuronal aging reflects a complex interplay between synaptic, metabolic, and electrophysiological changes rather than simple neuronal loss. In the context of the broader cellular networks of the brain, these neuronal alterations interact closely with age-related changes in glial and vascular populations, which modulate neuronal function and contribute to the evolving cellular landscape of the aging brain (Figure 1).

## 4 Glial reactivity and neuroinflammation

Glial cells are central regulators of brain homeostasis and play critical roles in shaping neuronal function, metabolic balance, and immune surveillance. During aging, however, glial populations undergo profound transcriptional, metabolic, and functional remodeling. These changes contribute to a shift from homeostatic support toward chronic low-grade inflammation, a phenomenon often described as neuroinflammaging (Müller et al., 2025a; Soraci et al., 2024).

Glial responses during aging encompass a spectrum of phenotypes that vary across brain regions, cellular subtypes, and environmental conditions. Evidence from human transcriptomic and spatial profiling studies indicates that aging-associated glial changes are regionally heterogeneous and involve altered inflammatory, metabolic, and phagocytic signaling pathways. Single-nucleus RNA sequencing analyses of aged human brains have identified reactive microglial and astrocytic states associated with inflammatory activation and neurodegenerative vulnerability (Olah et al., 2018; Gerrits et al., 2021). Importantly, glial cells do not act in isolation: microglia, astrocytes, and oligodendrocyte lineage cells form interconnected regulatory networks with neurons and vascular components that shape the trajectory of brain aging (Müller et al., 2025c; Hayashide et al., 2026; Carr et al., 2025).

### 4.1 Microglial priming and inflammaging

Microglia are the resident innate immune cells of the CNS and serve as the primary mediators of neuroimmune responses. Under physiological conditions, microglia continuously survey the brain parenchyma, clearing cellular debris, remodeling synapses, and supporting neuronal survival (Figure 1, right). Aging, however, profoundly alters microglial phenotype and function, leading to a state commonly referred to as microglial priming (Norden and Godbout, 2013).

Primed microglia display increased basal expression of pro-inflammatory cytokines and heightened responsiveness to secondary stimuli. Even in the absence of overt pathology, aged microglia show elevated levels of inflammatory mediators such as tumor necrosis factor (TNF), interleukin-1β (IL-1β), and interleukin-6 (IL-6), reflecting a chronic low-grade inflammatory state. This persistent inflammatory signaling contributes to the phenomenon of inflammaging, which is characterized by systemic and brain-specific increases in inflammatory mediators during aging (Soraci et al., 2024; Norden and Godbout, 2013; Woodburn et al., 2021; Masuda et al., 2019).

Mechanistically, several processes contribute to microglial priming. Aging microglia exhibit altered transcriptional programs involving immune signaling pathways such as NF-κB, reduced phagocytic efficiency, and accumulation of cellular waste due to impaired lysosomal degradation. These changes can lead to a progressive decline in the ability of microglia to maintain tissue homeostasis. In addition, aged microglia accumulate myelin debris resulting from ongoing myelin turnover, which can overload lysosomal pathways and promote cellular senescence (Müller and Di Benedetto, 2025a; Masuda et al., 2019; Kwon and Koh, 2020).

Recent single-cell transcriptomic studies have further revealed the emergence of distinct microglial states associated with aging. These include populations characterized by inflammatory signaling, altered lipid metabolism, and stress-response pathways. Some of these transcriptional profiles resemble microglial states observed in neurodegenerative diseases, suggesting that aging may predispose microglia toward disease-associated phenotypes (Wei and Li, 2022; Lauro and Limatola, 2020; Wendimu and Hooks, 2022; Valiukas et al., 2025; Colombo et al., 2022).

Importantly, microglial priming has significant consequences for neuronal circuits. Activated microglia release cytokines, chemokines, and ROS that can alter synaptic transmission, disrupt neuronal plasticity, and modulate astrocyte activation (Lana et al., 2016; Kabba et al., 2018). Microglia also interact with oligodendrocyte progenitor cells (OPCs), influencing myelin repair and regeneration. In aging, dysregulated microglial signaling may impair OPC differentiation and contribute to myelin degeneration, illustrating how microglial dysfunction can propagate pathology across multiple glial and neuronal compartments (Müller and Di Benedetto, 2025a; Benarroch, 2023; Fang and Bai, 2023).

### 4.2 Astrocyte reactivity and metabolic support

Astrocytes represent the most abundant glial population in the CNS and perform a wide range of functions essential for neuronal survival and synaptic activity (Figure 1, left). These include regulation of neurotransmitter clearance, maintenance of extracellular ion balance, modulation of synaptic plasticity, and metabolic support of neurons through the astrocyte–neuron metabolic coupling system (Müller et al., 2025c; Singh, 2022; Lee et al., 2022).

During aging, astrocytes undergo significant phenotypic changes that give rise to a spectrum of reactive states. Reactive astrocytes display altered gene expression patterns, hypertrophic morphology, and changes in the secretion of cytokines and growth factors. Importantly, astrocyte can adopt diverse functional states ranging from protective to neurotoxic phenotypes depending on the surrounding microenvironment (Liddelow et al., 2017; Clarke et al., 2018; Patani et al., 2023).

A key mechanism driving astrocyte reactivity involves signaling from activated microglia. Pro-inflammatory molecules such as TNF-α, IL-1α, and complement component C1q released by microglia can induce astrocytes to adopt a neuroinflammatory phenotype often referred to as A1-like reactive astrocytes. These astrocytes downregulate genes involved in synaptic support and begin to release inflammatory mediators and complement proteins that may contribute to neuronal damage and synapse loss (Lawrence et al., 2025).

Beyond their immune-related functions, astrocytes play a central role in metabolic support of neurons, particularly through the astrocyte–neuron lactate shuttle. Astrocytes metabolize glucose and provide lactate as an energy substrate for neurons during periods of high activity. Aging may disrupt this metabolic coupling by altering astrocytic glycolysis, mitochondrial metabolism, and substrate transport. Such metabolic changes can exacerbate neuronal energy deficits and contribute to age-related declines in synaptic function (Müller et al., 2025a; Müller and Di Benedetto, 2025a; Beard et al., 2021).

Astrocytes also interact closely with the cerebral vasculature through perivascular endfeet that regulate BBB integrity and neurovascular coupling. Consequently, astrocytic dysfunction during aging can affect vascular signaling, highlighting interactions between glial reactivity and neurovascular changes that are addressed in later sections (Knox et al., 2022; Sanmarco et al., 2021).

### 4.3 Myelin dynamics and oligodendrocyte aging

Oligodendrocytes and their progenitor cells, OPCs, are responsible for the formation and maintenance of myelin sheaths that insulate axons and facilitate rapid action potential conduction. Myelin is increasingly recognized as a dynamic structure that undergoes continuous remodeling throughout life. However, aging is associated with progressive disruptions in myelin maintenance and regeneration (Williamson and Lyons, 2018; Osso and Hughes, 2024). Structural studies have shown that aging leads to myelin fragmentation, thinning of myelin sheaths, and accumulation of myelin debris, particularly in white matter tracts and association fibers. These changes can impair axonal conduction velocity and disrupt synchronization within neural circuits, potentially contributing to cognitive slowing observed in aging populations (Huang et al., 2025; Groh and Simons, 2025).

At the cellular level, oligodendrocyte lineage cells exhibit age-related alterations in differentiation and regenerative capacity (Figure 1). OPCs persist in the adult brain and normally respond to demyelination by generating new oligodendrocytes. During aging, however, OPC proliferation and differentiation become less efficient, reducing the capacity for myelin repair. This decline may result from intrinsic changes in OPC transcriptional programs as well as extrinsic factors within the aging brain microenvironment (Mironova et al., 2026).

Interactions with other glial cells play an important role in regulating oligodendrocyte aging. Microglia participate in the clearance of myelin debris and release signals that influence OPC differentiation. However, in the aged brain, microglial dysfunction can impair this process and inhibit efficient remyelination. Similarly, astrocytes contribute to oligodendrocyte metabolism by supplying lipids and metabolic substrates required for myelin synthesis. Dysregulation of astrocytic metabolic pathways may therefore indirectly affect myelin maintenance during aging (Lopez-Muguruza and Matute, 2023; Fang and Bai, 2023; Matejuk et al., 2021).

Together, these findings illustrate that glial aging represents an interconnected network process involving immune activation, metabolic remodeling, and structural alterations in myelin integrity. Microglial priming, astrocyte reactivity, and oligodendrocyte dysfunction do not occur independently but rather interact dynamically with neuronal and vascular components of the brain. These complex glial responses play a critical role in shaping the inflammatory and metabolic landscape of the aging brain and contribute to the cellular environment in which neuronal aging and neurodegenerative processes unfold.

## 5 Vascular and neurovascular aging

**Figure 2:**
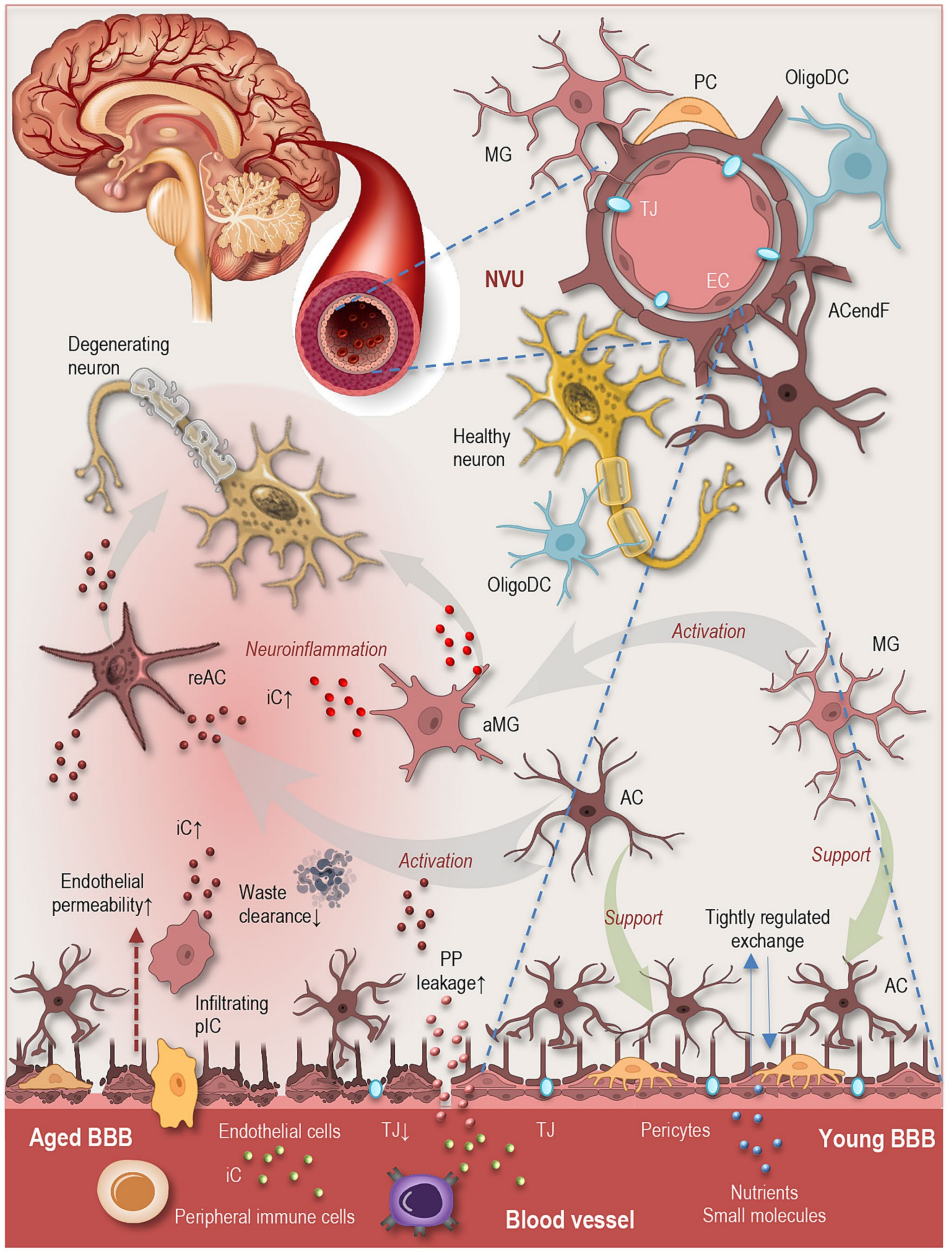
Brain aging: neurovascular unit organization and age-related BBB dysfunction. Simplified schematic illustrating the cellular architecture of the neurovascular unit and structural changes associated with aging. The NVU (upper right panel) is a highly integrated system composed of endothelial cells, pericytes, astrocytic endfeet, microglia, and neurons, which together regulate cerebral blood flow, metabolic exchange, and barrier function. The BBB (lower right panel) is primarily formed by specialized endothelial cells connected by tight junctions and supported by perivascular cells and astrocytic endfeet, creating a highly selective interface that tightly regulates the exchange of molecules between the circulation and the CNS. During aging (lower left panel), multiple structural and molecular alterations compromise BBB integrity. These include reduced expression of tight junction proteins, increased endothelial permeability, and impaired transport mechanisms regulating nutrient influx and waste clearance. Aging is also associated with increased leakage of plasma proteins into the brain parenchyma. Senescent endothelial cells exhibit altered metabolic and inflammatory profiles, diminished tight junction integrity, and weakened interactions with pericytes and astrocytes, collectively contributing to barrier dysfunction. Increased BBB permeability can expose neural tissue to circulating inflammatory mediators, immune cells, and plasma proteins, thereby promoting glial activation and neuronal dysfunction and linking vascular aging to neuroinflammatory processes. NVU, neurovascular unit; MG, microglia; PC, pericyte; OligoDC, oligodendrocyte; ACendF: astrocytic endfeet; TJ, tight junctions; EC, endothelial cell; MG, microglia; aMG, activated microglia; AC, astrocyte; reAC, reactive astrocyte; iC, inflammatory cytokines; PP, plasma proteins; pIC, peripheral immune cell; BBB, blood–brain barrier.

The cerebral vasculature plays a central role in maintaining brain homeostasis by regulating the delivery of oxygen, glucose, and circulating factors while simultaneously protecting neural tissue from potentially harmful blood-derived substances. These functions are mediated by the NVU—a highly integrated system composed of endothelial cells, pericytes, vascular smooth muscle cells, astrocytic endfeet, microglia, and neurons (Figure 2). With advancing age, structural and functional alterations within this system progressively impair vascular integrity and communication across cellular compartments, thereby contributing to neuronal dysfunction and increased susceptibility to neurodegenerative disease (Castro and Potente, 2022; Iadecola, 2017).

### 5.1 Blood–brain barrier disruption

One of the most prominent vascular changes associated with aging is the gradual decline in BBB integrity. The BBB is formed primarily by specialized endothelial cells connected by tight junctions and supported by perivascular cells and astrocytic endfeet (Figure 2). This structure tightly regulates the exchange of molecules between the circulation and the CNS, ensuring stable ionic conditions and protecting neural tissue from toxins and pathogens (Knox et al., 2022).

During aging, several structural and molecular changes compromise BBB function. These include reduced expression of tight junction proteins, increased endothelial permeability, and impaired transport mechanisms that regulate nutrient influx and waste clearance (Figure 2). Experimental studies in rodents have shown that aging is associated with increased leakage of plasma proteins into the brain parenchyma, indicating reduced barrier selectivity and compromised BBB integrity. Human neuroimaging and cerebrospinal fluid studies further suggest that BBB permeability increases with age, particularly within hippocampal regions associated with cognitive decline (Montagne et al., 2015). Age-related vascular dysfunction in humans has also been linked to impaired neurovascular coupling and reduced cerebral perfusion (Knox et al., 2022; Takata et al., 2021).

Recent work suggests that endothelial cell senescence may be a key driver of BBB deterioration. With age, these cells undergo molecular and functional changes characterized by oxidative stress, chronic low-grade inflammation, and reduced nitric oxide bioavailability. These alterations impair vasodilation and disrupt communication between vascular and neural cells (Graves and Baker, 2020; Csik et al., 2025). Senescent endothelial cells display altered metabolic and inflammatory profiles, reduced tight junction integrity, and diminished interactions with pericytes and astrocytes, collectively weakening the barrier function of cerebral microvessels (Graves and Baker, 2020).

Thus, endothelial dysfunction during aging may emerge not only as a vascular phenomenon but also as a network-level process involving bidirectional signaling between vascular and neural cell types (Neyra Chauca et al., 2026). Importantly, BBB disruption has consequences that extend beyond vascular pathology. Increased permeability can expose neural tissue to circulating inflammatory mediators, immune cells, and plasma proteins that promote glial activation and neuronal dysfunction, thereby linking vascular aging to neuroinflammatory processes.

### 5.2 Impairment of neurovascular coupling

Neurovascular coupling (NVC) refers to the dynamic process by which local neuronal activity triggers rapid increases in cerebral blood flow to meet metabolic demand. This process involves coordinated signaling between neurons, astrocytes, endothelial cells, and vascular smooth muscle cells. Aging can disrupt this finely tuned communication, leading to impaired matching between neuronal activity and vascular supply (Csipo et al., 2019).

Experimental studies in aged animal models demonstrate reduced NVC responses following sensory or neuronal stimulation, indicating diminished vascular responsiveness to neural signals. Mechanistically, these impairments may arise from astrocytic dysfunction, endothelial oxidative stress, or alterations in vasoactive signaling pathways such as nitric oxide and prostaglandin signaling (Csipo et al., 2019; Negri et al., 2026).

Human functional imaging studies also suggest that NVC becomes less efficient with age. Altered hemodynamic responses measured by functional MRI indicate that the vascular response to neuronal activation is delayed or attenuated in older adults, reflecting reduced vascular adaptability. Such changes can complicate the interpretation of neuroimaging signals and may contribute to decreased cognitive flexibility and processing efficiency in aging populations (Fesharaki et al., 2026; Zhao et al., 2021; Hu et al., 2025).

### 5.3 Neurovascular aging as a network process

Taken together, these findings highlight that vascular aging is not limited to structural changes within blood vessels but represents a broader systems-level transformation of the neurovascular unit. Endothelial dysfunction, BBB disruption, and reduced perfusion interact closely with glial reactivity and neuronal metabolic stress, creating feedback loops that can amplify cellular vulnerability across the brain (Takata et al., 2021). Understanding these interactions is therefore essential for interpreting brain aging as a multi-cellular network phenomenon, in which vascular components play a critical role in shaping neuronal resilience and susceptibility to neurodegenerative disease (Müller et al., 2025a; Müller and Di Benedetto, 2025a). In the following sections, we further examine how molecular stress pathways and inflammatory signaling intersect with these cellular interactions to influence the trajectory of brain aging.

## 6 Molecular programs driving cellular aging

Although neurons, glia, and vascular cells exhibit distinct aging trajectories, many of the underlying mechanisms are shared across cell types and reflect common molecular stress programs that accumulate over time. These processes—including mitochondrial dysfunction, impaired proteostasis, cellular senescence, DNA damage responses, and epigenetic remodeling—constitute interconnected hallmarks of cellular aging that progressively reshape the functional landscape of the brain (Zia et al., 2021). Understanding these shared molecular programs is therefore essential for interpreting brain aging as a coordinated multi-cellular process.

### 6.1 Mitochondrial dysfunction and metabolic decline

Mitochondria are central regulators of cellular energy metabolism, calcium homeostasis, and apoptotic signaling (Pandey, 2025). In the aging brain, mitochondrial function declines across multiple cell types, leading to reduced ATP production, increased generation of ROS, and impaired metabolic flexibility (Bondy, 2024; Srivastava, 2017; Jimenez-Loygorri et al., 2024).

Age-related changes in mitochondrial dynamics—including dysregulation of fusion, fission, and mitophagy—further contribute to the accumulation of damaged mitochondria within cells. Impaired mitochondrial quality control can exacerbate oxidative stress and disrupt cellular metabolism, creating a feedback loop that accelerates cellular aging. In glial cells, mitochondrial dysfunction may also influence inflammatory signaling pathways, thereby linking metabolic decline to neuroinflammation. Collectively, these processes highlight mitochondria as key regulators of cellular resilience and vulnerability during brain aging (Bondy, 2024; Chen and Chan, 2009).

### 6.2 Proteostasis impairment

Maintenance of protein homeostasis, or proteostasis, is essential for cellular function and survival. Proteostasis networks encompass protein synthesis, folding, trafficking, and degradation pathways, including the ubiquitin-proteasome system and autophagy-lysosomal pathways. During aging, these systems gradually lose efficiency, leading to the accumulation of misfolded or damaged proteins (Pandey, 2025; Shukla and Narayan, 2025).

In neurons, impaired proteostasis can disrupt synaptic function by altering the turnover of synaptic proteins and signaling molecules. Similarly, glial cells rely on efficient protein quality control mechanisms to maintain their diverse physiological roles, including immune surveillance and metabolic support. Dysfunction of autophagic and lysosomal pathways has been observed in aged microglia and astrocytes, potentially contributing to the accumulation of cellular debris and exacerbation of inflammatory signaling (Tseng et al., 2023; Hakim et al., 2016; Quick et al., 2023).

The decline of proteostasis is particularly relevant to neurodegenerative diseases, where pathological protein aggregates represent a defining feature. However, even in normal aging, subtle impairments in proteostasis can alter cellular signaling and stress responses, suggesting that protein quality control pathways represent a critical determinant of brain aging trajectories (Tseng et al., 2023).

### 6.3 Cellular senescence

Cellular senescence is characterized by a stable state of cell cycle arrest accompanied by profound changes in gene expression, metabolic activity, and secretory behavior. While this state plays important physiological roles in processes such as tissue repair and tumor suppression, the accumulation of senescent cells with age can contribute to tissue dysfunction (Pandey, 2025; Melo Dos Santos et al., 2024).

In the brain, senescence has been described in multiple cell types, including astrocytes, microglia, oligodendrocyte progenitor cells, and endothelial cells. Senescent cells typically adopt a senescence-associated secretory phenotype (SASP) characterized by the release of inflammatory cytokines, chemokines, proteases, and growth factors. This secretory profile can influence neighboring cells and alter the surrounding microenvironment, thereby amplifying inflammatory signaling and promoting cellular dysfunction across the NVU (Melo Dos Santos et al., 2024; Shafqat et al., 2023).

Recent evidence suggests that senescent glial cells may contribute to chronic neuroinflammation and impair regenerative processes such as remyelination and synaptic remodeling. Similarly, endothelial senescence has been implicated in BBB disruption and vascular dysfunction, linking cellular senescence to broader neurovascular alterations observed in aging (Shafqat et al., 2023; Hruby and Higuchi-Sanabria, 2025).

### 6.4 DNA damage responses

The accumulation of genomic damage represents another central driver of cellular aging. DNA lesions arise from multiple sources, including oxidative stress, replication errors, and environmental insults. Although cells possess sophisticated DNA repair mechanisms, the efficiency of these systems declines with age, leading to the gradual accumulation of genomic instability (Pandey, 2025; Delint-Ramirez and Madabhushi, 2025; Caldecott et al., 2022).

Neurons are particularly vulnerable to DNA damage due to their high metabolic activity and long lifespan. Persistent DNA lesions can disrupt transcriptional programs and impair neuronal function (Delint-Ramirez and Madabhushi, 2025). In glial and vascular cells, DNA damage can activate stress signaling pathways and inflammatory responses, further contributing to cellular dysfunction (Dash et al., 2025).

Activation of DNA damage response pathways can also promote cellular senescence and apoptosis, linking genomic instability to other hallmarks of aging. These processes may therefore act as upstream triggers that initiate broader molecular cascades affecting cellular homeostasis across the aging brain.

### 6.5 Epigenetic remodeling and transcriptional drift

Epigenetic mechanisms—including DNA methylation, histone modifications, and chromatin remodeling—play crucial roles in regulating gene expression and maintaining cellular identity. During aging, epigenetic landscapes undergo progressive alterations that can lead to transcriptional drift, characterized by increased variability and dysregulation of gene expression patterns (Pandey, 2025; la Torre et al., 2023).

Age-associated changes in DNA methylation patterns have been widely documented and form the basis of epigenetic clocks that estimate biological age across tissues. In the brain, epigenetic remodeling affects genes involved in synaptic function, immune signaling, and metabolic regulation. Such changes may influence the responsiveness of neurons and glial cells to environmental stimuli and stress signals (Lossi et al., 2024).

Importantly, epigenetic alterations can also interact with other molecular aging processes. For example, mitochondrial dysfunction and metabolic changes can influence chromatin-modifying enzymes through alterations in cellular metabolite levels, while DNA damage responses can trigger chromatin remodeling events. These interactions highlight the integrated nature of molecular aging pathways (Lossi et al., 2024; van der Rijt et al., 2020).

### 6.6 Convergence of molecular aging programs across cell types

Together, mitochondrial dysfunction, proteostasis decline, cellular senescence, genomic instability, and epigenetic remodeling form a network of interconnected processes that collectively drive cellular aging in the brain (Lopez-Otin et al., 2023). While each mechanism can affect specific cellular functions, their interactions may create systemic changes that influence neuronal signaling, glial activation, and vascular integrity. Importantly, these molecular programs can operate across multiple cell types, suggesting that brain aging arises from the integration of shared cellular stress pathways within complex cellular networks (Lopez-Otin et al., 2023; Glavan et al., 2026).

## 7 Systems-level integration of brain aging

While individual cellular and molecular mechanisms contribute to age-related changes in the brain, increasing evidence indicates that brain aging is fundamentally a systems-level phenomenon emerging from complex networks of interacting cells, signaling pathways, and metabolic processes. Neurons, glial populations, and vascular elements can form highly interconnected signaling systems within the NVU and broader brain microenvironment. With advancing age, alterations in cellular communication, metabolic coordination, and immune signaling may progressively reshape these networks, ultimately influencing neural circuit function and resilience to disease (Müller et al., 2025a,b; Zou et al., 2025; Müller and Di Benedetto, 2026). Understanding brain aging therefore requires integrative frameworks that capture multi-cellular interactions across spatial and molecular scales.

### 7.1 Intercellular signaling networks in the aging brain

Communication between brain cell types relies on complicated signaling networks involving neurotransmitters, cytokines, growth factors, metabolic intermediates, and extracellular vesicles. These signals enable neurons, glia, and vascular cells to coordinate metabolic supply, immune responses, and synaptic plasticity. During aging, however, the architecture of these signaling networks can undergo significant remodeling (Seguin et al., 2023; Di Benedetto et al., 2019; Chalmers et al., 2022).

Single-cell and spatial transcriptomic studies have revealed age-related changes in ligand–receptor signaling pathways across multiple cell types, indicating shifts in intercellular communication within the brain microenvironment (Li et al., 2023; Tsai et al., 2025). For example, alterations in cytokine signaling, complement pathways, and metabolic communication networks have been identified across neuronal and glial populations in aging tissues. These changes can influence synaptic regulation, immune activation, and tissue repair processes, highlighting how molecular signaling networks integrate cellular responses across the aging brain (Zheng et al., 2025).

Importantly, these signaling pathways often involve bidirectional and multidirectional interactions. Neurons influence microglial activation through neurotransmitter signaling and neuronal activity patterns, while glial cells regulate neuronal excitability and synaptic remodeling through cytokine and metabolic signaling. Astrocytes additionally coordinate metabolic exchange between neurons and blood vessels, linking neuronal activity to vascular responses. Such interconnected communication networks enable the brain to dynamically regulate its functional state but may also propagate dysfunction when regulatory mechanisms become impaired with age (Müller et al., 2025c; Zheng et al., 2025).

### 7.2 Immune–neural–vascular interactions

A central feature of systems-level brain aging is the close integration of immune, neural, and vascular signaling systems. The NVU provides a structural and functional framework for these interactions, integrating neurons, astrocytes, microglia, endothelial cells, pericytes, and vascular smooth muscle cells into a coordinated regulatory system. This unit controls cerebral blood flow, maintains BBB integrity, and modulates immune surveillance within the central nervous system (Iadecola, 2017; Neyra Chauca et al., 2026).

During aging, dysfunction in one component of this system can influence the entire network. For instance, endothelial dysfunction and impaired BBB integrity can permit the infiltration of peripheral immune factors into the brain, altering microglial activation and astrocytic signaling (Figure 2). Conversely, inflammatory mediators produced by activated microglia and astrocytes can influence vascular function, altering endothelial signaling pathways and vascular tone (Knox et al., 2022; Takata et al., 2021).

Recent work on neurovascular aging highlights how vascular cells themselves undergo transcriptional and functional remodeling that affects neuronal metabolism, immune trafficking, and the clearance of metabolic waste products from the brain. These vascular changes can compromise oxygen and glucose delivery, impair transport mechanisms across the BBB, and disrupt the clearance of potentially toxic proteins (Iadecola, 2017; Graves and Baker, 2020; Csik et al., 2025).

Through these mechanisms, age-related alterations in immune, neural, and vascular signaling pathways may converge to influence brain function at multiple levels, from cellular metabolism to large-scale neural network activity.

### 7.3 Feedback loops driving inflammaging

A key feature of systems-level brain aging is the emergence of self-reinforcing feedback loops that amplify inflammatory signaling across cellular networks. Chronic low-grade inflammation arises from the interaction of multiple molecular and cellular processes, including mitochondrial dysfunction, cellular senescence, DNA damage responses, and impaired immune regulation (Müller et al., 2025b; Voicu et al., 2025).

These processes create interconnected feedback loops. For example, mitochondrial dysfunction increases ROS production, which can activate inflammatory signaling pathways such as NF-κB. In turn, inflammatory mediators can further impair mitochondrial function and promote cellular senescence, reinforcing the inflammatory state. Similarly, senescent cells produce a pro-inflammatory secretome that affects neighboring cells and can modify the surrounding microenvironment (Müller et al., 2025a,c; Müller and Di Benedetto, 2026; Voicu et al., 2025).

Such interactions can propagate inflammation across cellular networks, influencing neurons, glia, and vascular cells simultaneously. As a result, inflammaging emerges not from a single molecular pathway but from network-level interactions between multiple cellular stress responses. Recent systems-level analyses of aging biology emphasize how mitochondrial dysfunction, DNA damage, and impaired mitophagy converge as central hubs connecting cellular aging mechanisms with inflammatory signaling pathways (Voicu et al., 2025).

### 7.4 Multiomics integration and systems biology approaches

The complexity of these interconnected processes has motivated the development of integrative approaches that combine diverse biological datasets to construct comprehensive models of brain aging. Advances in high-throughput technologies now allow simultaneous profiling of genomic, epigenomic, transcriptomic, proteomic, metabolomic, and lipidomic data from the same biological samples. Integrating these datasets through systems biology frameworks can reveal relationships between molecular pathways that may not be detectable using single-omics approaches (Cohen et al., 2022; Adewale et al., 2021; Vitorino, 2024).

Large-scale human multiomic and neuroimaging datasets increasingly support the concept that brain aging reflects coordinated alterations across interacting neuronal, glial, immune, and vascular systems rather than isolated cellular dysfunction. Integrative analyses combining transcriptomic, imaging, and biomarker data have begun to identify systems-level signatures associated with cognitive resilience and vulnerability during aging (Dong and Zhong, 2025; Ren et al., 2025; De Jager et al., 2018).

Systems biology tools—including network modeling, machine learning, and genome-scale metabolic reconstructions—are increasingly used to interpret these datasets. By constructing molecular interaction networks and identifying key regulatory nodes, these approaches allow researchers to map the hierarchical organization of biological processes that drive brain aging (Ren et al., 2025). Such models can also predict how perturbations in specific pathways may propagate through cellular networks to influence overall brain function.

### 7.5 Toward a network framework of brain aging

Taken together, these findings suggest that brain aging should be conceptualized not as a collection of independent cellular processes but as an emergent property of multi-layered biological networks. Intercellular signaling pathways, immune–vascular interactions, metabolic regulation, and gene expression programs form interconnected systems that operate across multiple spatial and temporal scales (Müller et al., 2025a; Müller and Di Benedetto, 2026).

Within this framework, resilience or vulnerability to aging-related decline may depend on the stability and adaptability of these networks (Müller et al., 2025a). Disruptions in key regulatory nodes—such as mitochondrial metabolism, immune signaling pathways, or vascular function—can propagate through cellular interactions and alter the functional organization of neural circuits.

By integrating molecular, cellular, and systems-level perspectives, network-based approaches provide a powerful framework for understanding the complexity of brain aging. Such approaches may ultimately enable the identification of critical network hubs and early biomarkers that can guide strategies to preserve brain function and promote healthy aging.

## 8 Outlook and future perspectives: towards tracing cellular aging trajectories

A major challenge in brain aging research is reconstructing the trajectories through which cellular and molecular changes unfold across the lifespan. Cross-sectional studies have provided valuable snapshots of age-related alterations but cannot capture the temporal dynamics of aging or distinguish causal mechanisms from adaptive responses. Future progress will therefore depend on integrative approaches combining longitudinal human studies, experimental cellular models, multiomic biomarker discovery, and interventions promoting healthy brain aging.

### 8.1 Longitudinal approaches to mapping brain aging

Longitudinal studies provide a critical framework for understanding how brain structure and function change over time. Repeated measurements within individuals allow researchers to distinguish true aging trajectories from stable interindividual differences (Jockwitz et al., 2021; Vinci-Booher et al., 2025). Advances in multimodal neuroimaging and computational modeling now enable the simultaneous assessment of structural, functional, and metabolic changes across aging populations, revealing that different brain features may follow partially independent temporal trajectories that influence cognitive outcomes (Vinci-Booher et al., 2025; Chen et al., 2026).

Large population cohorts such as the UK Biobank further support the development of normative models of brain aging. Machine-learning approaches applied to neuroimaging data have generated “brain age” metrics that estimate biological brain age from structural and functional features. Deviations between predicted and chronological age may indicate accelerated aging and increased disease risk (Zhang R. et al., 2025; Li et al., 2026).

### 8.2 Human stem cell and organoid models

Human induced pluripotent stem cells (iPSCs) and brain organoids provide powerful experimental platforms for studying cellular mechanisms of brain aging. iPSC-derived neurons, astrocytes, and microglia allow investigation of cell-type–specific processes such as mitochondrial dysfunction, proteostasis impairment, and inflammatory signaling (Mrza et al., 2024).

Organoid systems extend these approaches by reconstructing three-dimensional neural tissue environments that support neuronal–glial interactions and early circuit formation (Guo et al., 2025). Recent developments incorporate microglia and vascular-like components to model aspects of the neurovascular unit and intercellular communication networks. Although current organoid systems primarily resemble early developmental stages, strategies including prolonged culture and metabolic stress are being explored to induce aging-like phenotypes *in vitro* (Guo et al., 2025; O'Halloran et al., 2025).

### 8.3 Early biomarkers of brain aging

Identifying early biomarkers capable of predicting vulnerability to cognitive decline remains a major goal in aging research. Neuroimaging-derived measures—including brain age estimates, cortical thickness, and connectivity patterns—have emerged as promising indicators of biological brain aging (Yusri et al., 2024; Kocar et al., 2026). Complementary efforts are identifying circulating metabolic and inflammatory markers associated with brain aging phenotypes. Integrating imaging, molecular, and clinical data through multiomic frameworks may ultimately enable predictive signatures of brain aging and early identification of individuals at risk for neurodegenerative disease (Zwilling et al., 2024). Several longitudinal human cohort studies further suggest that combined imaging and circulating biomarker signatures may predict accelerated brain aging before overt clinical symptoms emerge (Cole and Franke, 2017; Franke and Gaser, 2019).

### 8.4 Interventions promoting healthy brain aging

Understanding cellular aging trajectories also creates opportunities for intervention. Lifestyle factors such as physical activity, diet, sleep regulation, and cardiovascular health are consistently associated with improved cognitive outcomes and reduced signatures of brain aging (Di Benedetto et al., 2017; Behrenbruch et al., 2026; Di Benedetto and Müller, 2019; Müller and Di Benedetto, 2025b).

At the molecular level, therapeutic strategies targeting mitochondrial dysfunction, chronic inflammation, and impaired proteostasis are under investigation in preclinical models (Delrue et al., 2025). In parallel, regenerative approaches—including stem cell therapies, gene editing technologies, and strategies aimed at rejuvenating neural stem cell populations—represent emerging avenues for restoring cellular function in the aging brain (Saliev and Singh, 2024; Joshi et al., 2025).

Taken together, future advances in brain aging research will likely arise from integrating longitudinal human studies, experimental cellular systems, and systems-level computational analyses. By combining multiomic data with predictive modeling frameworks, researchers may begin to trace cellular aging trajectories across biological scales—from molecular networks to neural circuits and whole-brain systems—ultimately enabling earlier detection and more effective prevention of age-related neurological disease.

## 9 Conclusion

The study of brain aging is undergoing a conceptual shift from viewing aging as a collection of independent cellular deficits toward understanding it as a *dynamic and interconnected systems process*. Insights from single-cell and spatial technologies now reveal that aging unfolds through diverse and asynchronous cellular trajectories that vary across neuronal, glial, and vascular populations as well as across brain regions. These discoveries challenge simplified models of uniform cellular decline and instead highlight the importance of *intercellular communication networks* in shaping how the aging brain adapts to physiological stressors.

Within this framework, aging can be understood as a gradual reorganization of cellular interactions that normally sustain brain homeostasis. Changes in neuronal activity, glial immune states, and vascular regulation influence each other through tightly coupled signaling pathways, metabolic exchange, and structural support systems. Disruptions in these relationships may propagate across multilayered cellular networks, amplifying inflammatory signaling, metabolic imbalance, and circuit dysfunction. Understanding these cross-cellular dynamics will be critical for explaining why certain brain regions and circuits remain resilient while others become vulnerable during aging.

Despite rapid progress, several key questions remain. How early do age-related cellular trajectory shifts begin, and which signals initiate them? Which intercellular signaling nodes act as critical regulators of resilience or vulnerability? And how do systemic factors—such as immune aging, metabolic state, or microbiome-derived signals—interact with local brain networks to shape aging outcomes? Addressing these questions will require integrative strategies that combine longitudinal human studies, advanced cellular models, and multiomic network analyses. Ultimately, deciphering how the cellular choreography of the brain evolves across the lifespan may reveal new opportunities to preserve the coordinated interactions that sustain neural resilience during aging.

## References

[ref1] Abrous D. N. Blin N. Boraxbekk C. J. Catheline G. Fitzsimons C. P. Hilscher M. . (2026). Hallmarks of healthy cognitive aging: inter-individual differences in aging trajectories. Ageing Res. Rev. 119:103102. doi: 10.1016/j.arr.2026.103102, 41865894

[ref2] Adewale Q. Khan A. F. Carbonell F. Iturria-Medina Y. Alzheimer's Disease Neuroimaging Initiative (2021). Integrated transcriptomic and neuroimaging brain model decodes biological mechanisms in aging and Alzheimer's disease. eLife 10:e62589. doi: 10.7554/eLife.62589 34002691 PMC8131100

[ref3] Ali M. A. Siam M. H. B. Vardaman D. 3rd Bolding C. Brazell J. N. Whatley A. D. . (2025). High-dimensional single-cell analysis reveals coordinated age-dependent neuroinflammatory microglia-T cell circuits in the brain. bioRxiv [Preprint]. doi: 10.64898/2025.12.10.693494

[ref4] Badimon A. Strasburger H. J. Ayata P. Chen X. Nair A. Ikegami A. . (2020). Negative feedback control of neuronal activity by microglia. Nature 586, 417–423. doi: 10.1038/s41586-020-2777-8, 32999463 PMC7577179

[ref5] Barberis M. Xie J. (2026). Spatial dynamics in health and disease: from neurodevelopment to therapeutic target identification for inflammatory diseases. Signal Transduct. Target. Ther. 11:49. doi: 10.1038/s41392-026-02589-5, 41656282 PMC12883626

[ref6] Barnes C. A. (1994). Normal aging: regionally specific changes in hippocampal synaptic transmission. Trends Neurosci. 17, 13–18. doi: 10.1016/0166-2236(94)90029-9, 7511843

[ref7] Barrantes F. J. (2024). Cognitive synaptopathy: synaptic and dendritic spine dysfunction in age-related cognitive disorders. Front. Aging Neurosci. 16:1476909. doi: 10.3389/fnagi.2024.1476909, 39420927 PMC11484076

[ref8] Beard E. Lengacher S. Dias S. Magistretti P. J. Finsterwald C. (2021). Astrocytes as key regulators of brain energy metabolism: new therapeutic perspectives. Front. Physiol. 12:825816. doi: 10.3389/fphys.2021.825816, 35087428 PMC8787066

[ref9] Behrenbruch N. Schwarck S. Schumann-Werner B. Molloy E. N. Garcia-Garcia B. Hochkeppler A. . (2026). A physically and mentally active lifestyle relates to younger brain and cognitive age. Geroscience 48, 1853–1873. doi: 10.1007/s11357-025-01764-w, 40619559 PMC12972277

[ref10] Benarroch E. (2023). What are the roles of oligodendrocyte precursor cells in Normal and pathologic conditions? Neurology 101, 958–965. doi: 10.1212/WNL.0000000000208000, 37985182 PMC10663025

[ref11] Bloss E. B. Janssen W. G. Ohm D. T. Yuk F. J. Wadsworth S. Saardi K. M. . (2011). Evidence for reduced experience-dependent dendritic spine plasticity in the aging prefrontal cortex. J. Neurosci. 31, 7831–7839. doi: 10.1523/JNEUROSCI.0839-11.2011, 21613496 PMC3398699

[ref12] Bondy S. C. (2024). Mitochondrial dysfunction as the major basis of brain aging. Biomolecules 14:402. doi: 10.3390/biom14040402, 38672420 PMC11048299

[ref13] Burke S. N. Barnes C. A. (2006). Neural plasticity in the ageing brain. Nat. Rev. Neurosci. 7, 30–40. doi: 10.1038/nrn1809, 16371948

[ref14] Burke S. Trudeau L. E. (2022). Axonal domain structure as a putative identifier of neuron-specific vulnerability to oxidative stress in cultured neurons. eNeuro 9, ENEURO.0139–ENEU22.2022. doi: 10.1523/ENEURO.0139-22.2022, 36192156 PMC9595591

[ref15] Cabeza R. Anderson N. D. Locantore J. K. McIntosh A. R. (2002). Aging gracefully: compensatory brain activity in high-performing older adults. NeuroImage 17, 1394–1402. doi: 10.1006/nimg.2002.1280, 12414279

[ref16] Caldecott K. W. Ward M. E. Nussenzweig A. (2022). The threat of programmed DNA damage to neuronal genome integrity and plasticity. Nat. Genet. 54, 115–120. doi: 10.1038/s41588-021-01001-y, 35145299

[ref17] Carr L. Mustafa S. Collins-Praino L. E. (2025). The hallmarks of ageing in microglia. Cell. Mol. Neurobiol. 45:45. doi: 10.1007/s10571-025-01564-y, 40389766 PMC12089641

[ref18] Castro M. Potente M. (2022). The blood-brain barrier-a metabolic ecosystem. EMBO J. 41:e111189. doi: 10.15252/embj.2022111189, 35437788 PMC9058533

[ref19] Chalmers R. A. Cervin M. Choo C. Baune B. T. Trollor J. N. Numbers K. . (2022). Networks of inflammation, depression, and cognition in aging males and females. Aging Clin. Exp. Res. 34, 2387–2398. doi: 10.1007/s40520-022-02198-6, 35895279 PMC9637618

[ref20] Chen H. Chan D. C. (2009). Mitochondrial dynamics--fusion, fission, movement, and mitophagy—in neurodegenerative diseases. Hum. Mol. Genet. 18, R169–R176. doi: 10.1093/hmg/ddp326, 19808793 PMC2758711

[ref21] Chen X. Yuan Z. Zhang J. Zhang X. Y. (2026). Multimodal quantitative MRI reveals age-related biophysical alterations in the human brain across the adult lifespan. NeuroImage 327:121742. doi: 10.1016/j.neuroimage.2026.121742 41570953

[ref22] Clarke L. E. Liddelow S. A. Chakraborty C. Munch A. E. Heiman M. Barres B. A. (2018). Normal aging induces A1-like astrocyte reactivity. Proc. Natl. Acad. Sci. USA 115, E1896–E1905. doi: 10.1073/pnas.1800165115, 29437957 PMC5828643

[ref23] Cohen A. A. Ferrucci L. Fulop T. Gravel D. Hao N. Kriete A. . (2022). A complex systems approach to aging biology. Nat. Aging 2, 580–591. doi: 10.1038/s43587-022-00252-6, 37117782 PMC12007111

[ref24] Cole J. H. Franke K. (2017). Predicting age using neuroimaging: innovative brain ageing biomarkers. Trends Neurosci. 40, 681–690. doi: 10.1016/j.tins.2017.10.001, 29074032

[ref25] Colombo G. Cubero R. J. A. Kanari L. Venturino A. Schulz R. Scolamiero M. . (2022). A tool for mapping microglial morphology, morphOMICs, reveals brain-region and sex-dependent phenotypes. Nat. Neurosci. 25, 1379–1393. doi: 10.1038/s41593-022-01167-6, 36180790 PMC9534764

[ref26] Csik B. Nyul-Toth A. Gulej R. Patai R. Kiss T. Delfavero J. . (2025). Senescent endothelial cells in cerebral microcirculation are key drivers of age-related blood-brain barrier disruption, microvascular rarefaction, and neurovascular coupling impairment in mice. Aging Cell 24:e70048. doi: 10.1111/acel.70048, 40167015 PMC12266767

[ref27] Csipo T. Mukli P. Lipecz A. Tarantini S. Bahadli D. Abdulhussein O. . (2019). Assessment of age-related decline of neurovascular coupling responses by functional near-infrared spectroscopy (fNIRS) in humans. Geroscience 41, 495–509. doi: 10.1007/s11357-019-00122-x, 31676966 PMC6885078

[ref28] Dash U. C. Bhol N. K. Swain S. K. Samal R. R. Nayak P. K. Raina V. . (2025). Oxidative stress and inflammation in the pathogenesis of neurological disorders: mechanisms and implications. Acta Pharm. Sin. B 15, 15–34. doi: 10.1016/j.apsb.2024.10.004, 40041912 PMC11873663

[ref29] De Domenico M. (2017). Multilayer modeling and analysis of human brain networks. Gigascience 6, 1–8. doi: 10.1093/gigascience/gix004, 28327916 PMC5437946

[ref30] De Jager P. L. Ma Y. McCabe C. Xu J. Vardarajan B. N. Felsky D. . (2018). A multi-omic atlas of the human frontal cortex for aging and Alzheimer's disease research. Sci. Data 5:180142. doi: 10.1038/sdata.2018.142, 30084846 PMC6080491

[ref31] Deery H. A. Di Paolo R. Moran C. Egan G. F. Jamadar S. D. (2023). Lower brain glucose metabolism in normal ageing is predominantly frontal and temporal: a systematic review and pooled effect size and activation likelihood estimates meta-analyses. Hum. Brain Mapp. 44, 1251–1277. doi: 10.1002/hbm.26119, 36269148 PMC9875940

[ref32] Delint-Ramirez I. Madabhushi R. (2025). DNA damage and its links to neuronal aging and degeneration. Neuron 113, 7–28. doi: 10.1016/j.neuron.2024.12.001, 39788088 PMC11832075

[ref33] Delrue C. Speeckaert R. Speeckaert M. M. (2025). Rewinding the clock: emerging pharmacological strategies for human anti-aging therapy. Int. J. Mol. Sci. 26:9372. doi: 10.3390/ijms26199372, 41096641 PMC12524491

[ref34] Di Benedetto S. Müller L. (2019). “Aging, immunity, and Neuroinflammation: the modulatory potential of nutrition,” in Nutrition and Immunity, eds. Mahmoudi M. Rezaei N. (Cham: Springer International Publishing), 301–322.

[ref35] Di Benedetto S. Müller L. Rauskolb S. Sendtner M. Deutschbein T. Pawelec G. . (2019). Network topology dynamics of circulating biomarkers and cognitive performance in older cytomegalovirus-seropositive or -seronegative men and women. Immun. Ageing 16:31. doi: 10.1186/s12979-019-0171-x, 31827568 PMC6894301

[ref36] Di Benedetto S. Müller L. Wenger E. Duzel S. Pawelec G. (2017). Contribution of neuroinflammation and immunity to brain aging and the mitigating effects of physical and cognitive interventions. Neurosci. Biobehav. Rev. 75, 114–128. doi: 10.1016/j.neubiorev.2017.01.044, 28161508

[ref37] Dong J. M. Zhong H. (2025). Systematic review: proteomics-driven multi-omics integration for Alzheimer's disease pathology and precision medicine. Neurol. Int. 17:197. doi: 10.3390/neurolint17120197, 41441216 PMC12736289

[ref38] Fang L.-P. Bai X. (2023). Oligodendrocyte precursor cells: the multitaskers in the brain. Pflugers Arch. - Eur. J. Physiol. 475, 1035–1044. doi: 10.1007/s00424-023-02837-5, 37401986 PMC10409806

[ref39] Fesharaki N. J. Taylor A. Ress D. (2026). Age-related variations of the hemodynamic response function spatially resolved across human cerebral cortex. Front. Aging Neurosci. 18:1774543. doi: 10.3389/fnagi.2026.1774543, 41822298 PMC12975896

[ref40] Franke K. Gaser C. (2019). Ten years of BrainAGE as a neuroimaging biomarker of brain aging: what insights have we gained? Front. Neurol. 10:789. doi: 10.3389/fneur.2019.00789, 31474922 PMC6702897

[ref41] Gerrits E. Brouwer N. Kooistra S. M. Woodbury M. E. Vermeiren Y. Lambourne M. . (2021). Distinct amyloid-beta and tau-associated microglia profiles in Alzheimer's disease. Acta Neuropathol. 141, 681–696. doi: 10.1007/s00401-021-02263-w 33609158 PMC8043951

[ref42] Glavan D. Doeppner T. R. Abuzan M. Hermann D. M. Capitanescu B. Olaru D. G. . (2026). Targeting the biology of aging in cerebrovascular disease: inflammation, metabolism, senescence, and regeneration. Int. J. Mol. Sci. 27:27(4). doi: 10.3390/ijms27041880, 41752015 PMC12941123

[ref43] Graves S. I. Baker D. J. (2020). Implicating endothelial cell senescence to dysfunction in the ageing and diseased brain. Basic Clin. Pharmacol. Toxicol. 127, 102–110. doi: 10.1111/bcpt.13403, 32162446 PMC7384943

[ref44] Groh J. Simons M. (2025). White matter aging and its impact on brain function. Neuron 113, 127–139. doi: 10.1016/j.neuron.2024.10.019 39541972

[ref45] Guo X. Wang X. Wang J. Ma M. Ren Q. (2025). Current development of iPSC-based modeling in neurodegenerative diseases. Int. J. Mol. Sci. 26:3774. doi: 10.3390/ijms26083774, 40332425 PMC12027653

[ref46] Hakim V. Cohen L. D. Zuchman R. Ziv T. Ziv N. E. (2016). The effects of proteasomal inhibition on synaptic proteostasis. EMBO J. 35, 2238–2262. doi: 10.15252/embj.201593594, 27613546 PMC5069550

[ref47] Ham S. Lee S. V. (2020). Advances in transcriptome analysis of human brain aging. Exp. Mol. Med. 52, 1787–1797. doi: 10.1038/s12276-020-00522-6, 33244150 PMC8080664

[ref48] Hammond T. R. Dufort C. Dissing-Olesen L. Giera S. Young A. Wysoker A. . (2019). Single-cell RNA sequencing of microglia throughout the mouse lifespan and in the injured brain reveals complex cell-state changes. Immunity 50, 253–271.e6. doi: 10.1016/j.immuni.2018.11.004, 30471926 PMC6655561

[ref49] Hayashide L. S. Pessoa B. Dias G. Pontes B. Pinto R. S. Diniz L. P. (2026). From neuron-centric to glia-centric: how aging glial networks drive neurodegenerative disease. J. Neurochem. 170:e70361. doi: 10.1111/jnc.70361 41591255 PMC12839804

[ref50] Head E. Corrada M. M. Kahle-Wrobleski K. Kim R. C. Sarsoza F. Goodus M. . (2009). Synaptic proteins, neuropathology and cognitive status in the oldest-old. Neurobiol. Aging 30, 1125–1134. doi: 10.1016/j.neurobiolaging.2007.10.001, 18006193 PMC7295175

[ref51] Henstridge C. M. Jackson R. J. Kim J. M. Herrmann A. G. Wright A. K. Harris S. E. . (2015). Post-mortem brain analyses of the Lothian birth cohort 1936: extending lifetime cognitive and brain phenotyping to the level of the synapse. Acta Neuropathol. Commun. 3:53. doi: 10.1186/s40478-015-0232-0, 26335101 PMC4559320

[ref52] Heuninckx S. Wenderoth N. Swinnen S. P. (2008). Systems neuroplasticity in the aging brain: recruiting additional neural resources for successful motor performance in elderly persons. J. Neurosci. 28, 91–99. doi: 10.1523/JNEUROSCI.3300-07.2008, 18171926 PMC6671150

[ref53] Hruby A. J. Higuchi-Sanabria R. (2025). Mitochondrial dysfunction in cellular senescence: a bridge to neurodegenerative disease. NPJ Aging 11:99. doi: 10.1038/s41514-025-00291-4, 41402339 PMC12708852

[ref54] Hu Y. Tao W. (2024). Current perspectives on microglia-neuron communication in the central nervous system: direct and indirect modes of interaction. J. Adv. Res. 66, 251–265. doi: 10.1016/j.jare.2024.01.006, 38195039 PMC11674795

[ref55] Hu Q. Zhang F. Wang H. Wang R. Zhang D. Fu R. . (2025). New mechanisms of aging: from vascular to neurological system. Brain Behav. Immun. Integr. 12:100143. doi: 10.1016/j.bbii.2025.100143

[ref56] Huang Z. Zhang Y. Zou P. Zong X. Zhang Q. (2025). Myelin dysfunction in aging and brain disorders: mechanisms and therapeutic opportunities. Mol. Neurodegener. 20:69. doi: 10.1186/s13024-025-00861-w, 40518508 PMC12168329

[ref57] Huuki-Myers L. A. Spangler A. Eagles N. J. Montgomery K. D. Kwon S. H. Guo B. . (2024). A data-driven single-cell and spatial transcriptomic map of the human prefrontal cortex. Science 384:eadh1938. doi: 10.1126/science.adh1938 38781370 PMC11398705

[ref58] Iadecola C. (2017). The neurovascular unit coming of age: a journey through neurovascular coupling in health and disease. Neuron 96, 17–42. doi: 10.1016/j.neuron.2017.07.030, 28957666 PMC5657612

[ref59] Jimenez-Loygorri J. I. Villarejo-Zori B. Viedma-Poyatos A. Zapata-Munoz J. Benitez-Fernandez R. Frutos-Lison M. D. . (2024). Mitophagy curtails cytosolic mtDNA-dependent activation of cGAS/STING inflammation during aging. Nat. Commun. 15:830. doi: 10.1038/s41467-024-45044-1, 38280852 PMC10821893

[ref60] Jockwitz C. Merillat S. Liem F. Oschwald J. Amunts K. Jancke L. . (2021). Generalizing longitudinal age effects on brain structure—a two-study comparison approach. Front. Hum. Neurosci. 15:635687. doi: 10.3389/fnhum.2021.635687, 33935669 PMC8085300

[ref61] Joshi J. M. Verma S. Seetharam R. N. Singh A. K. (2025). Stem cell-free therapy for healthy brain aging: mechanisms, challenges, and prospects. Biomed. Pharmacother. 192:118676. doi: 10.1016/j.biopha.2025.118676, 41130100

[ref62] Kabba J. A. Xu Y. Christian H. Ruan W. Chenai K. Xiang Y. . (2018). Microglia: housekeeper of the central nervous system. Cell. Mol. Neurobiol. 38, 53–71. doi: 10.1007/s10571-017-0504-2, 28534246 PMC11481884

[ref63] Knox E. G. Aburto M. R. Clarke G. Cryan J. F. O'Driscoll C. M. (2022). The blood-brain barrier in aging and neurodegeneration. Mol. Psychiatry 27, 2659–2673. doi: 10.1038/s41380-022-01511-z, 35361905 PMC9156404

[ref64] Kocar E. Sket R. Vasle A. H. Avgustin G. Benedik E. Seljak B. K. . (2026). Measuring biological age: insights from omics studies. Ageing Res. Rev. 114:102988. doi: 10.1016/j.arr.2025.102988, 41371352

[ref65] Kumar A. Bodhinathan K. Foster T. C. (2009). Susceptibility to calcium dysregulation during brain aging. Front. Aging Neurosci. 1:2. doi: 10.3389/neuro.24.002.2009, 20552053 PMC2874411

[ref66] Kwon H. S. Koh S.-H. (2020). Neuroinflammation in neurodegenerative disorders: the roles of microglia and astrocytes. Transl. Neurodegener. 9:42. doi: 10.1186/s40035-020-00221-2, 33239064 PMC7689983

[ref67] la Torre A. Lo Vecchio F. Greco A. (2023). Epigenetic mechanisms of aging and aging-associated diseases. Cells 12:1163. doi: 10.3390/cells12081163, 37190071 PMC10136616

[ref68] Lana D. Iovino L. Nosi D. Wenk G. L. Giovannini M. G. (2016). The neuron-astrocyte-microglia triad involvement in neuroinflammaging mechanisms in the CA3 hippocampus of memory-impaired aged rats. Exp. Gerontol. 83, 71–88. doi: 10.1016/j.exger.2016.07.011, 27466072

[ref69] Lauro C. Limatola C. (2020). Metabolic reprograming of microglia in the regulation of the innate inflammatory response. Front. Immunol. 11:493. doi: 10.3389/fimmu.2020.00493, 32265936 PMC7099404

[ref70] Lawrence J. M. Dampier W. Mell J. C. De Souza D. R. Schardien K. Yeakle K. . (2025). Inflammatory microglia signals drive A1-like polarization of astrocytes even in the presence of HIV-1 tat. Mol. Neurobiol. 63:251. doi: 10.1007/s12035-025-05409-z, 41345807 PMC12678590

[ref71] Lee J. Kim H. J. (2022). Normal aging induces changes in the brain and neurodegeneration progress: review of the structural, biochemical, metabolic, cellular, and molecular changes. Front. Aging Neurosci. 14:931536. doi: 10.3389/fnagi.2022.931536, 35847660 PMC9281621

[ref72] Lee H. G. Wheeler M. A. Quintana F. J. (2022). Function and therapeutic value of astrocytes in neurological diseases. Nat. Rev. Drug Discov. 21, 339–358. doi: 10.1038/s41573-022-00390-x, 35173313 PMC9081171

[ref73] Li Y. Gao H. Lin L. Wu Y. Zhu X. (2026). UK biobank-centric advances in brain age prediction: a comprehensive review. Rev. Neurosci. 37, 19–42. doi: 10.1515/revneuro-2025-0055, 40997331

[ref74] Li B. Li J. Li B. Ouchi T. Li L. Li Y. . (2023). A single-cell transcriptomic atlas characterizes age-related changes of murine cranial stem cell niches. Aging Cell 22:e13980. doi: 10.1111/acel.13980, 37681346 PMC10652347

[ref75] Li M. L. Wu S. H. Song B. Yang J. Fan L. Y. Yang Y. . (2022). Single-cell analysis reveals transcriptomic reprogramming in aging primate entorhinal cortex and the relevance with Alzheimer's disease. Aging Cell 21:e13723. doi: 10.1111/acel.13723, 36165462 PMC9649611

[ref76] Liddelow S. A. Guttenplan K. A. Clarke L. E. Bennett F. C. Bohlen C. J. Schirmer L. . (2017). Neurotoxic reactive astrocytes are induced by activated microglia. Nature 541, 481–487. doi: 10.1038/nature21029, 28099414 PMC5404890

[ref77] Lindenberger U. (2014). Human cognitive aging: Corriger la fortune? Science 346, 572–578. doi: 10.1126/science.1254403, 25359964

[ref78] Lopez-Muguruza E. Matute C. (2023). Alterations of oligodendrocyte and myelin energy metabolism in multiple sclerosis. Int. J. Mol. Sci. 24:12912. doi: 10.3390/ijms241612912, 37629092 PMC10454078

[ref79] Lopez-Otin C. Blasco M. A. Partridge L. Serrano M. Kroemer G. (2023). Hallmarks of aging: an expanding universe. Cell 186, 243–278. doi: 10.1016/j.cell.2022.11.001, 36599349

[ref80] Lossi L. Castagna C. Merighi A. (2024). An overview of the epigenetic modifications in the brain under normal and pathological conditions. Int. J. Mol. Sci. 25:3881. doi: 10.3390/ijms25073881, 38612690 PMC11011998

[ref81] Lovinger D. M. (2008). Communication networks in the brain: neurons, receptors, neurotransmitters, and alcohol. Alcohol Res. Health 31, 196–214. 23584863 PMC3860493

[ref82] Lupo G. Gaetani S. Cacci E. Biagioni S. Negri R. (2019). Molecular signatures of the aging brain: finding the links between genes and phenotypes. Neurotherapeutics 16, 543–553. doi: 10.1007/s13311-019-00743-2, 31161490 PMC6694319

[ref83] Marques S. Zeisel A. Codeluppi S. van Bruggen D. Mendanha Falcao A. Xiao L. . (2016). Oligodendrocyte heterogeneity in the mouse juvenile and adult central nervous system. Science 352, 1326–1329. doi: 10.1126/science.aaf6463, 27284195 PMC5221728

[ref84] Masuda T. Sankowski R. Staszewski O. Bottcher C. Amann L. Sagar . (2019). Spatial and temporal heterogeneity of mouse and human microglia at single-cell resolution. Nature 566, 388–392. doi: 10.1038/s41586-019-0924-x 30760929

[ref85] Matejuk A. Vandenbark A. A. Offner H. (2021). Cross-talk of the CNS with immune cells and functions in health and disease. Front. Neurol. 12:672455. doi: 10.3389/fneur.2021.672455, 34135852 PMC8200536

[ref86] Melo Dos Santos L. S. Trombetta-Lima M. Eggen B. Demaria M. (2024). Cellular senescence in brain aging and neurodegeneration. Ageing Res. Rev. 93:102141. doi: 10.1016/j.arr.2023.102141, 38030088

[ref87] Mironova Y. A. Dang B. Heo D. Xu Y. K. T. Hsu A. Y. von Eugenin Bernhardi J. . (2026). Myelin is repaired by constitutive differentiation of oligodendrocyte progenitors. Science 391:eadu2896. doi: 10.1126/science.adu2896, 41570153 PMC12997438

[ref88] Montagne A. Barnes Samuel R. Sweeney M. D. Halliday M. R. Sagare A. P. Zhao Z. . (2015). Blood-brain barrier breakdown in the aging human hippocampus. Neuron 85, 296–302. doi: 10.1016/j.neuron.2014.12.032 25611508 PMC4350773

[ref89] Morrison J. H. Baxter M. G. (2012). The ageing cortical synapse: hallmarks and implications for cognitive decline. Nat. Rev. Neurosci. 13, 240–250. doi: 10.1038/nrn3200, 22395804 PMC3592200

[ref90] Mrza M. A. He J. Wang Y. (2024). Integration of iPSC-derived microglia into brain organoids for neurological research. Int. J. Mol. Sci. 25:3148. doi: 10.3390/ijms25063148, 38542121 PMC10970489

[ref91] Müller L. Di Benedetto S. (2024). Aging brain: exploring the interplay between bone marrow aging, immunosenescence, and neuroinflammation. Front. Immunol. 15:1393324. doi: 10.3389/fimmu.2024.1393324, 38638424 PMC11024322

[ref92] Müller L. Di Benedetto S. (2025a). Neuroimmune crosstalk in chronic neuroinflammation: microglial interactions and immune modulation. Front. Cell. Neurosci. 19:1575022. doi: 10.3389/fncel.2025.1575022, 40260075 PMC12009833

[ref93] Müller L. Di Benedetto S. (2025b). Immunosenescence and inflammaging: mechanisms and modulation through diet and lifestyle. Front. Immunol. 16:1708280. doi: 10.3389/fimmu.2025.1708280, 41425546 PMC12711513

[ref94] Müller L. Di Benedetto S. (2026). Network rewiring in the aging immune system: from chronic inflammation to age-related pathologies. Cells 15:414. doi: 10.3390/cells15050414, 41827848 PMC12984825

[ref95] Müller L. Di Benedetto S. Müller V. (2025a). From homeostasis to neuroinflammation: insights into cellular and molecular interactions and network dynamics. Cells 14:54. doi: 10.3390/cells14010054, 39791755 PMC11720143

[ref96] Müller L. Di Benedetto S. Müller V. (2025b). Neuroimmune dynamics and brain aging: mechanisms and consequences. Front. Aging Neurosci. 17:1715045. doi: 10.3389/fnagi.2025.1715045, 41346440 PMC12673371

[ref97] Müller L. Di Benedetto S. Müller V. (2025c). The dual nature of neuroinflammation in networked brain. Front. Immunol. 16:1659947. doi: 10.3389/fimmu.2025.1659947, 40909282 PMC12404926

[ref98] Müller V. Jirsa V. Perdikis D. Sleimen-Malkoun R. von Oertzen T. Lindenberger U. (2019). Lifespan changes in network structure and network topology dynamics during rest and auditory oddball performance. Front. Aging Neurosci. 11:138. doi: 10.3389/fnagi.2019.00138, 31244648 PMC6580332

[ref99] Navakkode S. Kennedy B. K. (2024). Neural ageing and synaptic plasticity: prioritizing brain health in healthy longevity. Front. Aging Neurosci. 16:1428244. doi: 10.3389/fnagi.2024.1428244, 39161341 PMC11330810

[ref100] Negri S. Nyul-Toth A. Milan M. Troyano-Rodriguez E. Tavakol S. Ihuoma J. . (2026). A minimally invasive framework reveals region-specific cerebrovascular remodeling in aging using Intravital functional ultrasound imaging and ultrasound localization microscopy (fUS-ULM). Adv. Sci. 13:e10754. doi: 10.1002/advs.202510754, 40946188 PMC12767048

[ref101] Neyra Chauca J. M. Vazquez VanDyck M. Espinoza Santana A. Robles Martinez G. G. Romero Vega K. A. Garcia Quintana N. . (2026). Microvascular failure in the aging brain: converging pathways of oxidative stress, inflammation, and endothelial decline. Biomedicine 14:130. doi: 10.3390/biomedicines14010130, 41595665 PMC12839336

[ref102] Norden D. M. Godbout J. P. (2013). Review: microglia of the aged brain: primed to be activated and resistant to regulation. Neuropathol. Appl. Neurobiol. 39, 19–34. doi: 10.1111/j.1365-2990.2012.01306.x, 23039106 PMC3553257

[ref103] O'Halloran L. Akinsete O. Kogan A. L. Wrona M. Mahdi A. F. (2025). 3D in vitro blood-brain barrier models: recent advances and their role in brain disease research and therapy. Front. Pharmacol. 16:1637602. doi: 10.3389/fphar.2025.1637602 41126959 PMC12537694

[ref104] Ojaimi J. Masters C. L. Opeskin K. McKelvie P. Byrne E. (1999). Mitochondrial respiratory chain activity in the human brain as a function of age. Mech. Ageing Dev. 111, 39–47. doi: 10.1016/S0047-6374(99)00071-8, 10576606

[ref105] Olah M. Patrick E. Villani A. C. Xu J. White C. C. Ryan K. J. . (2018). A transcriptomic atlas of aged human microglia. Nat. Commun. 9:539. doi: 10.1038/s41467-018-02926-5, 29416036 PMC5803269

[ref106] Osso L. A. Hughes E. G. (2024). Dynamics of mature myelin. Nat. Neurosci. 27, 1449–1461. doi: 10.1038/s41593-024-01642-2, 38773349 PMC11515933

[ref107] Pacca-Corrêa J. B. L. Fernandes B. M. Siqueira M. Schafbenker R. Baumart G. J. Damico I. V. . (2026). Astrocyte states in brain aging and neurodegeneration: at the crossroads of senescence and reactivity. Neurochem. Res. 51:102. doi: 10.1007/s11064-026-04709-7, 41803335 PMC12971753

[ref108] Pandey K. B. (2025). From bench to bedside: translational insights into aging research. Front. Aging 6:1492099. doi: 10.3389/fragi.2025.1492099, 39926027 PMC11802818

[ref109] Pass T. Wiesner R. J. Pla-Martin D. (2021). Selective neuron vulnerability in common and rare diseases-mitochondria in the focus. Front. Mol. Biosci. 8:676187. doi: 10.3389/fmolb.2021.676187, 34295920 PMC8290884

[ref110] Patani R. Hardingham G. E. Liddelow S. A. (2023). Functional roles of reactive astrocytes in neuroinflammation and neurodegeneration. Nat. Rev. Neurol. 19, 395–409. doi: 10.1038/s41582-023-00822-1, 37308616

[ref111] Pessoa B. Hayashide L. S. Dias G. Pontes B. Pinto R. S. Diniz L. P. (2026). Senescent astrocytes: a new player in brain aging and cognitive decline. Brain Sci. 16:76. doi: 10.3390/brainsci16010076 41594798 PMC12839264

[ref112] Quick J. D. Silva C. Wong J. H. Lim K. L. Reynolds R. Barron A. M. . (2023). Lysosomal acidification dysfunction in microglia: an emerging pathogenic mechanism of neuroinflammation and neurodegeneration. J. Neuroinflammation 20:185. doi: 10.1186/s12974-023-02866-y, 37543564 PMC10403868

[ref113] Radulescu C. I. Pilch K. S. Wang X. Gibbs F. Barnes S. J. (2025). Turning back time: aging plasticity and its rejuvenation. Curr. Opin. Neurobiol. 94:103097. doi: 10.1016/j.conb.2025.103097, 40829306

[ref114] Ren F. Wei J. Chen Q. Hu M. Yu L. Mi J. . (2025). Artificial intelligence-driven multi-omics approaches in Alzheimer's disease: Progress, challenges, and future directions. Acta Pharm. Sin. B 15, 4327–4385. doi: 10.1016/j.apsb.2025.07.030, 41049729 PMC12491694

[ref115] Saliev T. Singh P. B. (2024). From bench to bedside: translating cellular rejuvenation therapies into clinical applications. Cells 13:2052. doi: 10.3390/cells13242052, 39768144 PMC11674796

[ref116] Sanmarco L. M. Polonio C. M. Wheeler M. A. Quintana F. J. (2021). Functional immune cell-astrocyte interactions. J. Exp. Med. 218:e20202715. doi: 10.1084/jem.20202715, 34292315 PMC8302447

[ref117] Seguin C. Sporns O. Zalesky A. (2023). Brain network communication: concepts, models and applications. Nat. Rev. Neurosci. 24, 557–574. doi: 10.1038/s41583-023-00718-5, 37438433

[ref118] Shafqat A. Khan S. Omer M. H. Niaz M. Albalkhi I. AlKattan K. . (2023). Cellular senescence in brain aging and cognitive decline. Front. Aging Neurosci. 15:1281581. doi: 10.3389/fnagi.2023.1281581, 38076538 PMC10702235

[ref119] Shukla M. Narayan M. (2025). Proteostasis and its role in disease development. Cell Biochem. Biophys. 83, 1725–1741. doi: 10.1007/s12013-024-01581-6, 39422790 PMC12123047

[ref120] Singh D. (2022). Astrocytic and microglial cells as the modulators of neuroinflammation in Alzheimer's disease. J. Neuroinflammation 19:206. doi: 10.1186/s12974-022-02565-0, 35978311 PMC9382837

[ref121] Song I. Dityatev A. (2018). Crosstalk between glia, extracellular matrix and neurons. Brain Res. Bull. 136, 101–108. doi: 10.1016/j.brainresbull.2017.03.003, 28284900

[ref122] Soraci L. Corsonello A. Paparazzo E. Montesanto A. Piacenza F. Olivieri F. . (2024). Neuroinflammaging: a tight line between normal aging and age-related neurodegenerative disorders. Aging Dis. 15, 1726–1747. doi: 10.14336/AD.2023.1001 38300639 PMC11272206

[ref123] Srivastava S. (2017). The mitochondrial basis of aging and age-related disorders. Genes (Basel) 8:398. doi: 10.3390/genes8120398, 29257072 PMC5748716

[ref124] Sun E. D. Nagvekar R. Pogson A. N. Brunet A. (2025). Brain aging and rejuvenation at single-cell resolution. Neuron 113, 82–108. doi: 10.1016/j.neuron.2024.12.007, 39788089 PMC11842159

[ref125] Takata F. Nakagawa S. Matsumoto J. Dohgu S. (2021). Blood-brain barrier dysfunction amplifies the development of neuroinflammation: understanding of cellular events in brain microvascular endothelial cells for prevention and treatment of BBB dysfunction. Front. Cell. Neurosci. 15:661838. doi: 10.3389/fncel.2021.661838, 34588955 PMC8475767

[ref126] Theurey P. Pizzo P. (2018). The aging mitochondria. Genes (Basel) 9:22. doi: 10.3390/genes9010022, 29315229 PMC5793175

[ref127] Tran M. N. Maynard K. R. Spangler A. Huuki L. A. Montgomery K. D. Sadashivaiah V. . (2021). Single-nucleus transcriptome analysis reveals cell-type-specific molecular signatures across reward circuitry in the human brain. Neuron 109, 3088–3103.e5. doi: 10.1016/j.neuron.2021.09.001, 34582785 PMC8564763

[ref128] Tsai A. P. Henze D. E. Ramirez Lopez E. Haberberger J. Dong C. Lu N. . (2025). Spatial and single-cell transcriptomics reveal the reorganization of cerebellar microglia with aging. Cell Rep. 44:116624. doi: 10.1016/j.celrep.2025.116624, 41307999 PMC12904544

[ref129] Tseng C. S. Chao Y. W. Liu Y. H. Huang Y. S. Chao H. W. (2023). Dysregulated proteostasis network in neuronal diseases. Front. Cell Dev. Biol. 11:1075215. doi: 10.3389/fcell.2023.1075215, 36910151 PMC9998692

[ref130] Tsintzou A. Poirier R. Harati R. Kustubayeva A. Disdier C. Hamoudi R. . (2025). Bridging regional neurovascular unit heterogeneity and cognitive function: a review. Fluids Barriers CNS 22:85. doi: 10.1186/s12987-025-00697-y, 40836329 PMC12366203

[ref131] Valiukas Z. Tangalakis K. Apostolopoulos V. Feehan J. (2025). Microglial activation states and their implications for Alzheimer's disease. J. Prev Alzheimers Dis. 12:100013. doi: 10.1016/j.tjpad.2024.100013, 39800461 PMC12184064

[ref132] van der Rijt S. Molenaars M. McIntyre R. L. Janssens G. E. Houtkooper R. H. (2020). Integrating the hallmarks of aging throughout the tree of life: a focus on mitochondrial dysfunction. Front. Cell Dev. Biol. 8:594416. doi: 10.3389/fcell.2020.594416, 33324647 PMC7726203

[ref133] Villarreal D. M. Do V. Haddad E. Derrick B. E. (2002). NMDA receptor antagonists sustain LTP and spatial memory: active processes mediate LTP decay. Nat. Neurosci. 5, 48–52. doi: 10.1038/nn776, 11740500

[ref134] Vinci-Booher S. Ren X. Kay K. Yu C. Pestilli F. Booth J. R. (2025). Dense longitudinal neuroimaging reveals individual brain change trajectories. Trends Cogn. Sci. 30, 464–476. doi: 10.1016/j.tics.2025.09.005, 41058412 PMC12667233

[ref135] Vitorino R. (2024). Transforming clinical research: the power of high-throughput omics integration. Proteomes 12:25. doi: 10.3390/proteomes12030025, 39311198 PMC11417901

[ref136] Voicu V. Toader C. Serban M. Covache-Busuioc R. A. Ciurea A. V. (2025). Systemic neurodegeneration and brain aging: multi-omics disintegration, proteostatic collapse, and network failure across the CNS. Biomedicine 13:2025. doi: 10.3390/biomedicines13082025, 40868276 PMC12383969

[ref137] Wei Y. Li X. (2022). Different phenotypes of microglia in animal models of Alzheimer disease. Immun. Ageing 19:44. doi: 10.1186/s12979-022-00300-0, 36209099 PMC9547462

[ref138] Wendimu M. Y. Hooks S. B. (2022). Microglia phenotypes in aging and neurodegenerative diseases. Cells 11:2091. doi: 10.3390/cells11132091, 35805174 PMC9266143

[ref139] Williamson J. M. Lyons D. A. (2018). Myelin dynamics throughout life: an ever-changing landscape? Front. Cell. Neurosci. 12:424. doi: 10.3389/fncel.2018.00424, 30510502 PMC6252314

[ref140] Woodburn S. C. Bollinger J. L. Wohleb E. S. (2021). The semantics of microglia activation: neuroinflammation, homeostasis, and stress. J. Neuroinflammation 18:258. doi: 10.1186/s12974-021-02309-6, 34742308 PMC8571840

[ref141] Ximerakis M. Lipnick S. L. Innes B. T. Simmons S. K. Adiconis X. Dionne D. . (2019). Single-cell transcriptomic profiling of the aging mouse brain. Nat. Neurosci. 22, 1696–1708. doi: 10.1038/s41593-019-0491-3, 31551601

[ref142] Yusri K. Kumar S. Fong S. Gruber J. Sorrentino V. (2024). Towards healthy longevity: comprehensive insights from molecular targets and biomarkers to biological clocks. Int. J. Mol. Sci. 25:6793. doi: 10.3390/ijms25126793, 38928497 PMC11203944

[ref143] Zhang X. Gao Y. Zhang S. Wang Y. Pei X. Chen Y. . (2025). Mitochondrial dysfunction in the regulation of aging and aging-related diseases. Cell Commun. Signal 23:290. doi: 10.1186/s12964-025-02308-7, 40537801 PMC12177975

[ref144] Zhang D. Rubio Rodríguez-Kirby L. A. Lin Y. Wang W. Song M. Wang L. . (2025). Spatial dynamics of brain development and neuroinflammation. Nature 647, 213–227. doi: 10.1038/s41586-025-09663-y, 41193846 PMC12589135

[ref145] Zhang R. Yi F. Mao H. Huang Z. Wang K. Zhang J. (2025). Brain age gap as a predictive biomarker that links aging, lifestyle, and neuropsychiatric health. Commun. Med. (Lond.) 5:441. doi: 10.1038/s43856-025-01100-5, 41136538 PMC12552503

[ref146] Zhao Y. Liu P. Turner M. P. Abdelkarim D. Lu H. Rypma B. (2021). The neural-vascular basis of age-related processing speed decline. Psychophysiology 58:e13845. doi: 10.1111/psyp.13845, 34115388

[ref147] Zheng Y. Huang R. Pan J. (2025). Dynamic intercellular networks in the CNS: mechanisms of crosstalk from homeostasis to neurodegeneration. Int. J. Mol. Sci. 26:8155. doi: 10.3390/ijms26178155, 40943076 PMC12428373

[ref148] Zia A. Pourbagher-Shahri A. M. Farkhondeh T. Samarghandian S. (2021). Molecular and cellular pathways contributing to brain aging. Behav. Brain Funct. 17:6. doi: 10.1186/s12993-021-00179-9, 34118939 PMC8199306

[ref149] Zong Y. Li H. Liao P. Chen L. Pan Y. Zheng Y. . (2024). Mitochondrial dysfunction: mechanisms and advances in therapy. Signal Transduct. Target. Ther. 9:124. doi: 10.1038/s41392-024-01839-8, 38744846 PMC11094169

[ref150] Zou H. Zhang S. Cui X. Xu H. Zhou Z. Cheng D. . (2025). Advancements in the investigation of the mechanisms underlying cognitive aging. Biogerontology 26:158. doi: 10.1007/s10522-025-10300-4, 40783909 PMC12336085

[ref151] Zwilling C. E. Wu J. Barbey A. K. (2024). Investigating nutrient biomarkers of healthy brain aging: a multimodal brain imaging study. NPJ Aging 10:27. doi: 10.1038/s41514-024-00150-8, 38773079 PMC11109270

